# Illustration of patient-reported outcome challenges and solutions in rare diseases: a systematic review in Cushing’s syndrome

**DOI:** 10.1186/s13023-018-0958-4

**Published:** 2018-12-19

**Authors:** Naomi Knoble, Gabrielle Nayroles, Cherry Cheng, Benoit Arnould

**Affiliations:** 1 Mapi, an ICON plc Company, 27 rue de la Villette, 69003 Lyon, France; 2 Ipsen Pharma, 65 quai Georges Gorse, 92560 Boulogne-Billancourt, France

**Keywords:** Quality of life, Patient-reported outcome, Literature review, Rare disease, Cushing’s syndrome

## Abstract

Rare diseases are often not fully understood and efforts put in investigating it from patient perspective are usually met with challenges. We performed a systematic literature review (SLR) for the last 20 years in Cushing’s Syndrome (CS) to illustrate Patient-Reported Outcome (PRO) challenges, and show what solutions were found.

PROs and other Clinical Outcome Assessment (COA) used with CS patients were reviewed in 36 studies. Two CS-specific Health Related Quality of Life (HRQL) measures were identified (i.e., CushingQoL, Tuebingen CD-25), as well as depression and neurocognitive measures. For CS-specific HRQL measures, the CushingQoL was the most widely used measure due in part to being the first CS-specific HRQL measure developed. With algorithms mapping the CushingQoL to both the SF-6D and EQ-5D, the CushingQoL could be used to facilitate economic modelling studies in the absence of a generic HRQL measure. While the CushingQoL offers only the global scale and two subscales compared to the six subscales of the Tuebingen CD-25, there is not yet adequate statistical validation data available for the Tuebingen CD-25 to suggest it can withstand the scrutiny of review by multiple stakeholders. Results of this review indicate that the inclusion of a measure of depressive symptoms, such as the BDI-II or similar measure, would be reasonable to include given the high level of comorbidity of depression among CS patients. A brief neurocognitive performance outcome, such as Trail Making tasks A and D or Digit Symbol, could help inform the interpretation of HRQL results. Neurocognitive differences may be an unassessed mediator of HRQL outcomes, partly accounting for the persistence of depressive symptoms and HRQL deficits despite treatment. Results suggest that HRQL improvements are possible within this population. These results are limited by small sample sizes and pre/post study design.

CS showcases the difficulties encountered in measuring PROs in rare diseases. A solution for this specific case was developed in the form of dedicated PRO instruments, the CushingQOL and the Tuebingen-25. However, some aspects of CS may not be fully answered or not yet validated (e.g., depressive and cognitive symptoms). Further research needs to be done to address them.

**Electronic supplementary material:**

The online version of this article (10.1186/s13023-018-0958-4) contains supplementary material, which is available to authorized users.

## Background

In Europe, a rare disease is defined as rare when it affects fewer than 1 in 2000, and in the US, a rare disease is defined as affecting less than 200,000 individuals [1]. Altogether, rare diseases affect 350 million people [2]. There are between 6000 to 8000 rare diseases with an estimation of 7% of the global population affected by one at one point in their life [3]. Despite this large number, insufficient resources are mobilized. Rare diseases are often not well understood, with patients suffering from isolation (e.g., only a few cases in one country), lack of information (e.g., few specialized healthcare practitioners), and limited access to medical care (e.g., specialized centre far away). The journey from disease discovery to reliable treatment and therapies can be very long. For example, Cushing’s syndrome (CS) was discovered almost a century ago, and still today patients do not fully recover from premorbid state after cure [4].

Health-Related Quality of Life (HRQL) and Patient-Reported Outcomes’ (PRO) usefulness have been recognized for understanding the impact of treatment on patient functioning and well-being [5]. HRQL and PRO may be used to evaluate and communicate the benefits of new drugs and devices from patient point of view, and efforts have been made to formalize them in the last decades [6–8]. In 2009, a guidance was issued by the Food and Drug Administration (FDA) to review and evaluate existing, modified or newly created PRO instruments used to support claims in approved medical product labelling [8]. However, the guidance does not address disease specific issues leaving the development of PRO tools, especially for rare disease, in a difficult position to follow guidelines. Indeed, the very nature of rare disease make it difficult to gather enough data at the right timing (e.g., few patients, patient’s availability, speed of disease’s progression), and often too few studies are led to better understand them. Moreover, existing and generic PRO instruments may miss crucial data, as they may not be sensitive enough to grasp a rare disease’s specificity.

CS, a hormonal disorder caused by prolonged exposure on body’s tissue to high levels of cortisol, is a rare disease affecting children and adults (from 4 to 80 years old) and having an incidence of less than one case per million per year [9]. The most common cause of CS is exogenous, and is caused by prolonged exposure to glucocorticoids, which are used to treat inflammatory diseases. Other causes are endogenous: 70% of times by pituitary adenomas, 15% by adrenal tumors, and 15% by non-pituitary tumors (ectopic ACTH secretion) [3]. Most people affected by CS experience upper body obesity, a rounded face, increased fat around the neck with slender limbs, easily bruised skin, weakened bones, wide purple striae, excess body hair and menstrual irregularities for women, and decreased fertility in men [1]. Along with physical problems, patients experience psychiatric and psychological disturbances (e.g., major depression, mania, anxiety disorders, and cognitive impairment). The condition has profound effect on patient’s quality of life (QOL), impairing areas such as body image, relations with family, friends and partner as well as work performance or school [10]. Current treatments for CS depend on specific reasons for excess cortisol [3], and may include surgery, radiation, cortisol-inhibiting drugs or, in extremely rare cases, chemotherapy. Even after curing, patients show poor general well-being and overall QOL, and remain anxious and depressed [4]. Assessment of HRQL is therefore salient for patient with CS, however with the challenges mentioned earlier.

The aim of this paper is to investigate and describe PRO measurement challenges, and what has been done to address this issue by conducting a targeted literature review, in the specific context of CS as a concrete example of a rare disease.

## Methods

### Search

We conducted a systematic literature review (SLR) to investigate PRO measurement in CS to meet the following objectives: review and synthesize evidence related to PRO measurement strategies in clinical trials with CS; identify optimal PROs for use in this disease area, and identify measurement challenges encountered in this specific disease.

The literature review on CS was conducted on articles published up to December 15th 2016 on the following sources: Medline database through Pubmed; Medline in Process; Ovid including Embase; Cochrane Central Register of Controlled Trial; PsycINFO; and Google Scholar for additional articles found in literature reviews. We used indexed search terms, including MeSH-indexed terms. Similar terms were used for each search on different database, according to requirements for each search engine. The terms used in this literature review were the following: “adrenocortical hyperplasia, acth induced”; “corticotropin induced adrenocortical hyperplasia”; “cushing syndrome, acth induced”; “Cushing’s disease”; “Cushings disease”; “itsenko cushing disease”; “pituitary ACTH hypersecretion”; “health related quality of life”; “HRQL”; “HRQoL”; “life quality”. Our initial intent was to select only clinical trials, but as the search did not retrieve many clinical trials, the search was expanded to other study designs (e.g., literature review, cross-sectional studies, etc.). Replicate of the research with the terms “ACTH independent” and “ectopic cushing syndrome” did not yield additional findings.

### Selection process

Titles and abstracts identified through the different database searches were independently screened by two reviewers following selection criteria (CS, English-language only, related to PRO or HRQL) to determine their inclusion for full-text review. Two reviewers then reviewed articles identified by this process for eligibility. Studies were excluded for the following reasons: not within disease area, non-english language, case study design, population out of scope (e.g., non-human subjects), and secondary paper. There was no restriction for year of publication. After the completion of both processes by each reviewer, any discrepancies between them were resolved through discussion. Every step of this process was documented and monitored in a Microsoft Excel grid, including reasons for exclusion.

### Data extraction

Data was extracted for all included articles and congress presentations for five domains: study design, patient characteristics, treatment, outcomes, and PRO measures (Table 1). Two independent reviewers performed data extraction, and discrepancies were resolved through discussion.

**Table 1:** Specific information collected within each domain of interest

Domains	Information collected within each domain
Study design	Study design; investigational interventions; study endpoints; inclusion criteria; exclusion criteria
Patient characteristics	Number of participants at baseline; main intervention; diagnosis; surgery
Treatment	Main intervention; dosage of medications; frequency of taking medications; treatment duration
Outcomes	Cushing QoL; Tuebingen CD-25; other HRQL measures utilized in the study; HRQOL factors; HRQL results; survival (number of patients who died during the study), response (how patients responded to treatment); healthcare utilization (length of hospital stays, visits to emergency room, unplanned medical visits)
PRO measures	PRO purpose; PRO administration (how it was used to collect patient data); PRO scoring; PRO factors (clinical areas assessed by the measure); PRO primary citation (original authors of the measure); reliability/validity of the PRO measure; number of items in the measure; limitations of the PRO measure; HRQL change (improvements or declines in functioning that was assessed using the measure); HRQL response change (external or environmental factors that impacts the patient’s HRQL); recommendations (comments on the usefulness of the measure)

*PRO* Patient-Reported Outcome, *HRQL* Health-Related Quality of Life; CushingQOL and Tuebingen CD-25 are PRO used in Cushing Syndrome

## Results

### Results from the literature searches

**Fig. 1:**
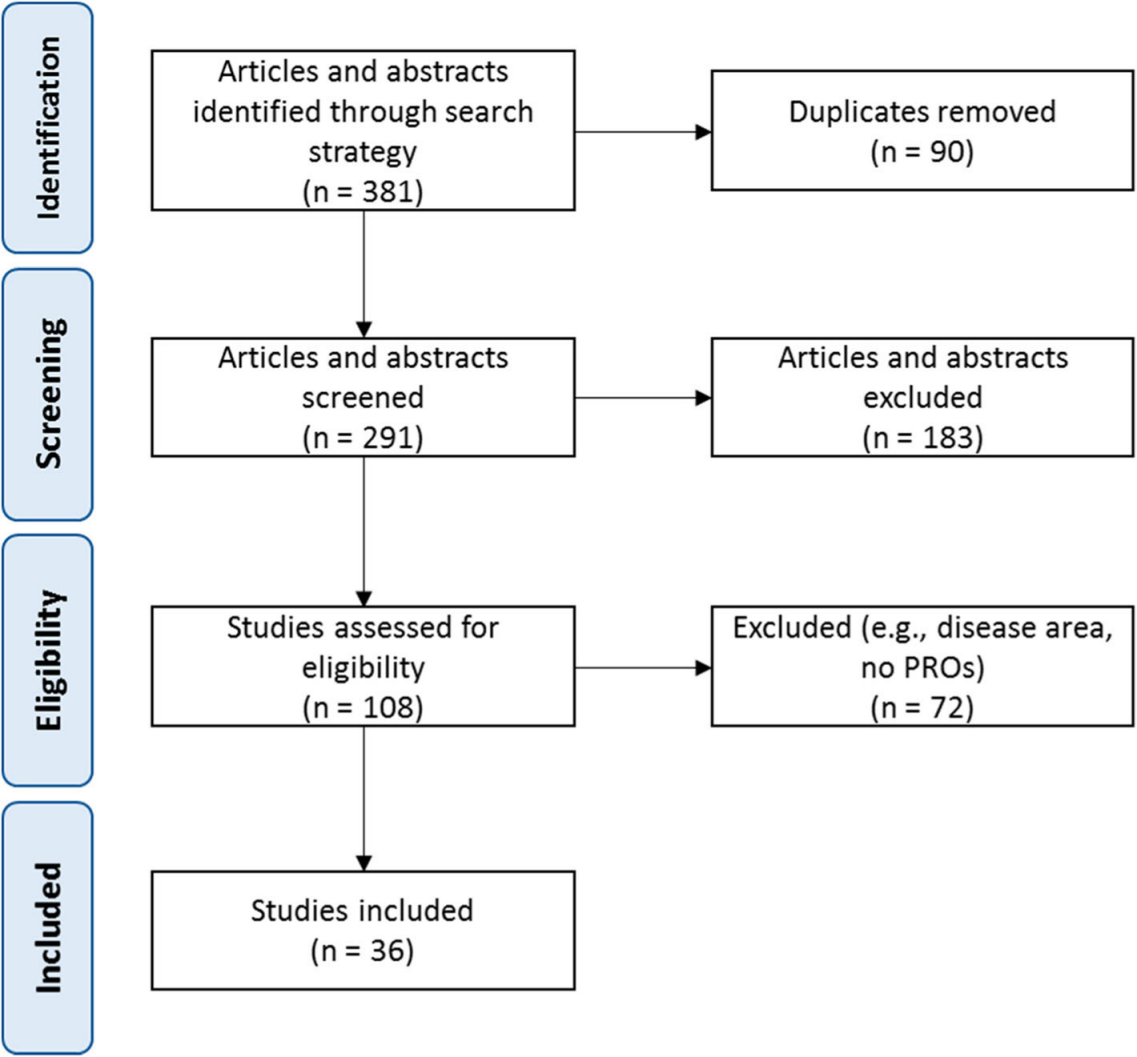
Selection process of the systematic literature review

The initial search yielded 381 titles and abstracts (3 from Cochrane Central Register of Controlled Trial; 7 from PsycINFO; 96 from Medline; and 275 from Embase). After removal of duplicates, 291 titles and abstracts were screened and 183 remained. After screening, 108 full-text articles were assessed for eligibility. In the end, 36 titles and abstracts specific to CS were identified and included in the review. Figure 1 summarizes the flow of articles through the selection process.

Only limited number of studies that were retrieved in this SLR were from clinical trials. We decided to include other study designs (e.g., literature reviews, cross-sectional studies, etc.) to document which PROs have been used in CS to identify measurement challenges.

Below is the summary of the findings from the literature review of the different biomedical literature databases. It is organized by study design: literature reviews; clinical trials; longitudinal observational studies; cross-sectional studies; utility value studies; and retrospective and case-controlled studies.

### Literature review

Eight literature reviews were identified in the search, addressing QOL [4, 10–12], paediatric patients [13], and neurocognitive and psychological functioning [14–16].

#### Quality of life for paediatric and adult CS patients

For both paediatric and adult CS patients, associated HRQL impairments are evident, and despite CS cure impairments persist and may never fall within normal ranges [11–13]. Two CS-specific PROs have been developed to measure HRQL, the CushingQoL and the Tuebingen CD-25 [4, 10]. As the Tuebingen CD-25 was more recently developed than the CushingQoL, relatively fewer studies have used it. Disease-related factors associated with impaired HRQL scores among CS patients identified with both CS-specific HRQL PROs appear to include urinary free cortisol (UFC) levels (reflecting greater intensity of hypercorticolism), length of time since CS diagnosis (indicating longer exposure to hypercorticolism), which may be due to a long delay to diagnosis, and depression. Evidence supporting demographic factors (e.g., age, sex) remains unclear and may be sample-dependent [10].

#### Neurocognitive and psychological functioning

Three studies have demonstrated the negative impact of excessive cortisol exposure on neurocognitive and psychological functioning [14–16]. Research indicates that depression is a common comorbid condition among CS patients prevalent in about 50 to 81% of CS patients, followed by anxiety disorders (e.g., generalized anxiety, panic disorder) with a prevalence of about 66% of patients, and mania with evidence indicating that despite long-term cure CS patients do not completely return to normal ranges of functioning [15, 16]. Existing evidence from cross-sectional research suggests that only depression independently accounts for HRQL outcomes among CS patients ( [17] as cited in [16]).

Results of 19 magnetic resonance imaging (MRI) studies summarized in a systematic literature review indicated that active CS was associated with smaller hippocampal volume, enlarged ventricles, and lower white and gray matter volume (i.e., cerebral atrophy) [14]. Results also indicated that following treatment of excess cortisol, neurocognitive structures did not completely revert to normal ranges. Indeed verbal learning, verbal memory, and mood functioning including severe depression were associated with structural and functional brain differences [14]. Severe depressive symptoms were observed in patients with long-term CS remission who also had reduced white matter in the left uncinated fasiculus (a region implicated in limbic system functioning) [14].

### Clinical trials

Four clinical trial studies were reviewed including one study from a pasireotide trial [18] and three from the SEISMIC trial [19–21]. Clinical outcome assessments (COA) used across these four studies included the 36-item short form survey of quality of life (QOL; SF-36) [21], CushingQoL [18, 19], Beck Depression Inventory (BDI) [18, 20], and Trail Making A and B tests (TMT A and B), a brief performance outcome (PerfO) of neurocognitive processing speed and executive functioning [20].

#### Pasireotide trial

In a randomized, double-blind, phase III study involving 162 persistent/recurrent or de novo CS patients, who were treated with Pasireotide 600 μg and 900 μg subcutaneous twice daily, results from the CushingQoL and BDI were reported. Timing of the BDI assessments was not reported. HRQL was assessed at baseline and months 3, 6 and 12 using the 12-item CushingQoL questionnaire. Clinically meaningful change in CushingQoL score was estimated to be > 10.1. Overall HRQL improved for 76 patients who had a second evaluation a year later, from a mean score of 41.1 at baseline (*N* = 159) to 52.5 at month 12 (*N* = 76; mean increase: 11.1; 95% CI: 6.8, 15.5). In both dose groups, HRQL improved with rapid and sustained decreases in urine free cortisol (UFC) levels. Twenty-nine patients whose UFC levels were controlled had a clinically meaningful improvement in HRQL at 12 months (mean improvement: 12.8; 95% CI: 7.1, 18.5). Results are similar for 17 partially UFC controlled patients (mean improvement: 10.7; 95% CI: 0.8, 20.5). However, the improvement in HRQL did not reach the specified 10.1 change threshold in the 30 patients uncontrolled group (mean improvement: 9.9; 95% CI: 2.3, 17.6). The highest HRQL improvements (≥20 points) were seen in the five patients with the largest UFC decreases (from >10xupper limits of normal to ≤5xupper limits of normal). Significant moderate negative correlations were detected between changes in the CushingQoL scores and changes in mean UFC (*r* = − 0.40; *n* = 68); as well as, the BDI score (*r* = − 0.59, *n* = 72), (*p* < 0.01). In addition, statistically significant mild positive correlations were observed between CushingQoL HRQL improvements and body mass index (BMI) and weight (*r* = 0.32, *n* = 74). A notable limitation of these findings is the significant amount of missing data from baseline to month 12 as results for 83 out of 159 patients were not reported [18].

#### SEISMIC trial and extension

In a SEISMIC trial study, a 24-week, open-label safety and efficacy trial of mifepristone, 50 patients who were included in the trial had endogenous CS [20]. Administered COAs included the BDI version 2 (BDI-II), TMT, and SF-36. At 24-weeks, for patients with at least mild depression at baseline (BDI-II scores of 14 or greater) median BDI-II depression scores improved from 23 (range 14–49) to 12 (range 0–34) in the modified intent-to-treat group, *p* < 0.001. These scores indicate an overall improvement falling within the minimal to no depression range (a BDI-II score of zero to 12 indicates minimal depression). However, unlike mild depression patients the range of scores for patients within the severe depression range at 24-weeks indicates a persisting depression burden. Cognition scores derived from the TMT improved on the Trail A, a measure of cognitive efficiency (median decrease of 4.0 s, *p* < 0.01), and Trail B, a measure of executive functioning (median decrease of 12 s, p < 0.01). The SF-36 scores significantly improved on the mental composite score (Mean = 40.0, SD = 14.5 vs. Mean = 45.4, SD = 12.5, *p* = 0.01) and the physical composite score (Mean = 34.9, SD = 11.0 vs. Mean = 39.1, SD = 10.8, *p* = 0.02) [19, 20].

In another SEISMIC report of data from 46 out of the 50 CS patients, HRQL as measured by the SF-36 was reported after taking mifeprestone [21]. At 24 weeks, statistically significant (*p* ≤ 0.05) improvements in HRQL from baseline were reported for the following subscales: general health, (Mean = 4.4, SD = 8.3, *p* = 0.004); physical function (Mean = 7.1, SD = 9.4, *p* < 0.0001); role physical (Mean = 3.3, SD = 10.4, *p* = 0.05); social functioning (Mean = 7.7, SD = 11.6, *p* = 0.0003); vitality (Mean = 6.3, SD = 11.1, *p* = 0.002); mental health (Mean = 4.1, SD = 10.5, *p* = 0.03); role emotional (Mean = 4.9, SD = 12.4, *p* = 0.03) which denotes progressive clinical improvement in patients’ physical appearance and QoL enhancement [21].

In a report of the open label follow-up extension study from the SEISMIC trial, HRQL was measured with the CushingQoL among 23 patients with persistent or recurring CS [19]. CushingQoL assessments were administered prior to beginning the SEISMIC study and during the extension study 6 weeks later. Change in score was calculated as % change over baseline. These twenty-three patients improved their item scores by 52% from the baseline, (*p* < 0.001), and statistically significant improvements between baseline and the extension study were shown in the following areas: bruising (86%, *p* = 0.037), socialization (74%, *p* < 0.001), physical appearance (73%, *p* < 0.001), sleep (59%, *p* = 0.001), mood swings (54%, *p* = 0.005), wound healing (52%, *p* = 0.002), desire for leisure activities (50%, p < 0.001), illness impact on activities of daily living (45%, *p* = 0.027), worries about future health (45%, p = 0.027), pain (44%, p = 0.037) and confidence (44%, *p* = 0.003). Notable limitations of these findings include sampling representativeness (i.e., only 23 out of the original 50 patients in the SEISMIC trial consented to the CushingQoL administration at the 3 months study visit following extension study entry), small sample size, and the unclear report of assessment timing.

### Longitudinal studies

Five longitudinal studies were included in this review including a study of pediatric patients [22], a focus on cognitive functioning [22, 23], pre/post-surgical intervention [24], a prospective survey [25], and a psychosocial intervention [26]. PROs and COAs used included HRQL measures (e.g., CushingQoL, SF-36), neurocognitive PerfOs (e.g., Wechsler Abbreviated Scale of Intelligence), the Child Health Questionnaire, symptom checklists (e.g., SCL-90), and other psychosocial measures. While all five studies capture patient experiences over varying lengths of time, all five used a pre/post measurement design.

Three studies directly measured HRQL and observed improvements for treated CS patients over time [24–26]. Among CS patients assessed over 7 years pre/post-adrenalectomy, significant mental and physical HRQL improvements as measured by the SF-36 were observed for treated patients with no significant correlations for age, sex, or length of follow-up [24]. In a survey follow-up study of post-adrenalectomy patients (mean length of follow-up was approximately 3 years), 78% of patients reported HRQL improvements on a single survey item assessing improvement, no change or worsening in HRQL [25]. In a 9-month long patient health education intervention study, CS patients (the majority of whom were in remission) who received the intervention had significantly better HRQL scores on the CushingQoL than the control group (Mean = 56.47, *SD* = 19.18 vs. Mean = 48.49, *SD* = 20.02, *p* < 0.01) resulting in improved physical activity, healthy lifestyle, better sleep patterns?. However, there were no significant differences in HRQL scores within the intervention group [26]. Significant correlations were observed between the CushingQoL score and reduced pain (*r* = 0.46, *p* < .05), improved physical activity (*r* = 0.89, *p* < .001), and sleep (*r* = 0.53, *p* < .05). There was a significant health resource utilization reduction observed for the intervention group, specifically a reduction in unscheduled visits and use of emergency services [26].

Neurocognitive functioning among children and adults was measured for CS patients pre/post treatment. Results suggested that older age at first evaluation for children was a protective factor against CS while a shorter duration of CS was protective among adults [22, 23]. In the paediatric study [22], it is likely that older age at first evaluation served as a proxy indicator for older age at CS diagnosis. Neurocognitive performance among children declined following treatment after 12-months yet still fell within the normal range [22]. Among adult patients followed over 12-months post-treatment, neurocognitive symptoms were observed to generally improve for CS patients, but at varying rates [23]. In this study, CS patient performance on the Digit Symbol task, a brief neurocognitive PerfO subscale that assessed cognitive efficiency and executive functioning, varied significantly by duration of CS when depressive symptoms were controlled. The magnitude of these findings was not reported.

### Cross-sectional studies

Thirteen cross-sectional studies were included in this review [17, 27–38]. Studies focused on neurocognitive functioning and HRQL among CS patients.

#### Neurocognitive functioning

Two cross-sectional studies examined neurocognitive functioning among CS patients and found that brain volume and executive functioning were associated with HRQL as measured by CushingQoL and a self-administered mental fatigue scale [27, 31]. In an MRI study of brain volume among CS patients and healthy controls, CS patients had lower overall brain volume and the volume of the right cerebellar cortex was positively associated with HRQL scores as measured by the CushingQoL [31]. In an examination of CS patient mental fatigue and executive functioning, results indicated that CS patients evidenced significantly greater mental fatigue (a proxy for HRQL) as well as greater executive dysfunction in the Trail Making extension D, a task with a relatively greater neurocognitive burden compared to tests A, B, and C, when compared to controls [27]. Of note, this study found that TMT A, B, and C did not differentiate patients from controls.

#### HRQL

Eleven of the cross-sectional studies reviewed examined HRQL among CS patients using the CushingQoL as a CS-specific questionnaire [17, 28–30, 32–38].

A number of studies examined HRQL within CS patient subgroups. Results from European Register on CS studies indicated no HRQL differences observed on the CushingQoL and EQ-5D-VAS scores between males and females or among the four etiologic groups (i.e., pituitary dependent CS, adrenal-dependent CS, ectopic source, other) [35–37]. A comparison between Cushing’s disease (CD) and CS patients was made in a large cross-sectional study validating the CushingQoL two-subscale solution (i.e., psychosocial, physical) with results indicating no differences among CD and CS patients on the subscales [34]. A study examining differences among CS patients in remission from pituitary and adrenal CS no differences were observed between CS groups on HRQL questionnaires, including the CushingQoL; however, it was observed that HRQL for CS patients in long-term remission remains impaired compared to controls [37].

A number of cross-sectional studies identified factors associated with HRQL level among CS patients. Three studies using CushingQoL observed a statistically significant positive association between longer CS remission duration and higher HRQL [28, 34, 37]. Additional research also examined the factors of time from CS symptoms to diagnosis, specifically early diagnosis, and receiving regular follow-up from a Cushing specialist and found significant positive associations with higher HRQL scores [28, 29].

Within the CushingQoL two-subscale solution study, the CushingQoL questionnaire demonstrated good known-group validity with lower psychosocial, physical, and global QOL results in patients with hypopituitarism compared to patients without hypopituitarism (psychosocial: Mean = 39.7, SD = 23 vs Mean = 48.8, SD = 23, respectively, Mann–Whitney U (U) = 14,280, *p* = 0.001; physical: Mean = 44.2, SD = 25 vs Mean = 56.1, SD = 22, respectively, U = 14,757.5, *p* < 0.001; global: Mean = 40.9, SD = 22 vs Mean = 50.7, SD = 21, U = 14,621, *p* < 0.001) and hydrocortisone use compared to no hydrocortisone use (psychosocial: Mean = 35.6, SD = 22 vs. Mean = 50.7, SD = 22, U = 18,452.5, p < 0.001; physical: Mean = 47.8, SD = 24 vs Mean = 56.9, SD = 22, U = 17,847.5, p < 0.001; global: Mean = 37.3, SD = 21 vs. Mean = 52.2,SD = 20, U = 18,711, p < 0.001) [34]. Hypopituitarism was also found to be a significant HRQL predictor in another study [28].

Additional HRQL factors examined among cross-sectional studies included illness perception (e.g., beliefs about the disease, cognitive and emotional disease impacts, sense of personal control) measured with the Illness Perception Questionnaire-Revised (IPQ-R) [32, 33]. Results from two studies by the same research team examining illness perception among CS patients indicated significant positive correlations with physical and emotional functioning of EQ-5D-VAS (physical symptom checklist *r* = 0.625, mobility *r* = 0.327, activity *r* = 0.329, anxiety *r* = 0.319) and negative mild and moderate correlations with global scales of EQ-5D-VAS: *r* = − 0.382 and CushingQoL: *r* = − 0.659 [32]. Interpretive projective measures were also used [33].

### Utility values studies

Two utility values studies conducted with different CS samples using the CushingQoL were found in this review [39, 40]. In order to facilitate cost-utility and other economic modeling studies, prediction models of preference-adjusted health status for CS patients were derived from the SF-36 (SF-6D) [39] and the EQ-5D [40] using individual items and the overall global score from the CushingQoL. Of note, depression and hospitalizations during the previous year were reportedly statistically associated with CushingQoL items in the final model (R^2^ = 0.65) [39]. These studies resulted in provisional models that provided algorithms for mapping the CushingQoL to the SF-6D and the EQ-5D, which could facilitate preference studies with the CushingQoL in the absence of data from these generic HRQL measures.

### Retrospective and case-controlled studies

Findings from four studies comprising retrospective chart review and case-controlled designs indicated that CS patient HRQL is lower than non-CS patients, and that CS patient HRQL may remain impaired following treatment [9, 41–43]. In a case-controlled study of a CS sample treated with laparoscopic bilateral adrenalectomy and a control group, a CS-specific questionnaire designed by the study team measured physical features of Cushing’s, biochemical abnormalities and comorbidities, and emotional-behavioural features of the disease and found that CS patients had significantly higher scores across all domains compared to the control group [42]. Using the SF-36, it was observed that HRQL was lowest among post-operative “cured” CS patients compared to patients with adrenal adenoma and persistent hypercortisolism [9]. In a retrospective chart review study followed by a case-control study, it was observed that HRQL as measured by the SF-36 did not fall within the normal range after long-term control of hypercortisolism among CS patients [41]. A retrospective survey study was conducted with CS patients regarding functioning pre/post treatment and results indicated that many CS patients experience persisting symptoms post-treatment [43].

## Discussion

### Patient-reported outcomes and others clinical outcome assessments (COA) in CS

#### CS-specific HRQL measures

The US Food and Drug Administration industry PRO guidance for labeling claims [8] indicates that a sounded measure must demonstrate usefulness within the specific patient population. Within CS, there are two prominent HRQL measures specifically designed for this patient population: Tuebingen CD-25 and CushingQoL. As the CushingQoL was the first HRQL measure developed for this population there are comparatively more studies that have used it, including within clinical trials, than the Tuebingen CD-25. The Tuebingen CD-25 was developed in 2011 and demonstrated good psychometric properties (high reliability: Cronbach’s alpha = 0.93; and validity: *r* = − 0.65) [44]. It was statistically validated against the CushingQoL in 2015 with a reasonable correlation (Spearman’s coefficient = − 0.73; *p* < 0.01) [45]. It includes 6 subscales: depression, sexual activity, environment, eating behaviour, bodily restrictions, cognition and a total score. The relative breadth of studies that have used the CushingQoL could facilitate comparison between future clinical trials and other trials in this disease area. The CushingQoL questionnaire shows good test-retest reliability, is valid, and shows better sensitivity to change than generic questionnaire such as the EQ-5D in real clinical practice [17, 46].

Further, two utility value studies were conducted with the CushingQoL resulting in algorithms mapping the CushingQoL to the SF-6D and EQ-5D. While NICE guidelines indicate that it is preferred to have results directly from the EQ-5D for calculating quality adjusted life years (QALYs), when EQ-5D data are not available mapping can be considered acceptable [47]. These two utility value studies could strengthen the rationale for using the CushingQoL as a CS-specific HRQL measure over the Tuebingen CD-25 as the available algorithms could facilitate economic modeling studies.

While the majority of studies included in this SLR used the CushingQoL, the data reported from this measure primarily used the global scale. The use of the CushingQoL in clinical trials prior to the development of the two subscale scoring solution that was validated for this measure is likely to explain the few results with these subscales so far [34]. The two-subscale scoring method for psychosocial and physical HRQL may increase the specificity of HRQL domains measured by this instrument. For more information about the HRQoL measures used in each reference cited, please see Additional file 1 and Additional file 2.

#### Depressive symptoms

Given the prevalence of depression within the CS patient population, the inclusion of a PRO specific to depressive symptoms in a clinical trial appears to be indicated. The BDI-II was used across clinical trials. While the BDI-II is a brief, widely used and well-validated screening instrument for depression, a significant limitation is that it is only available in US English and Spanish. The Tuebingen CD-25 includes a depression subscale, yet this subscale has not yet been validated with other PROs for depressive symptoms and warrants additional research.

#### Neurocognitive functioning

In some circumstances, neurocognitive and psychological functioning can have an impact on a patient QOL. Hence, taking into account this matter is important when developing a PRO instrument. Results indicate that neurocognitive functioning is impacted by CS and may affect HRQL. In other diseases, such as traumatic brain injury and multiples sclerosis, there is evidence of a moderate correlation between physical function and HRQoL, as the two dimensions are linked but not redundant [48, 49]. A PRO measure to assess neurocognitive functioning can be confounded by the degree of neurocognitive impairment the patient has experienced. Due to this confounding, the inclusion of a neurocognitive PerfO may be warranted. Among other clinical trials, the brief TMT A and B were used with statistically significant improvements observed following treatment. It should be noted that there are four tasks for Trail Making: A, B, C, and D. Trail Making D is a task with a relatively greater neurocognitive burden compared to tasks A, B, and C. Within one of the cross-sectional studies Trail Making D differentiated CS patients from controls while Trail Making A, B, and C did not [27]. The use of TMT, either A and B or A and D, may be useful for a brief measure of CS patients’ neurocognitive functioning. Trail Making does not require special qualifications for the individual administering the task.

An additional neurocognitive PerfO task, Digit Symbol, was used within one of the longitudinal studies. Digit Symbol is a brief, time-limited neurocognitive task similar to the Trail Making PerfO used in the SEISMIC trial study [20]. Although the Digit Symbol task places greater neurocognitive demands on patients than Trail Making A and B, Digit Symbol can be a relatively brief PerfO assessment that does not require special qualifications for the individual administering the task, but certification and quality control is required for training in order to administer the task.

Although TMT and Digit Symbol PerfOs are brief to administer, they are potentially resource and time-intensive to administer. These PerfOs require administration by an individual, monitoring and timing the patient during the administration, and scoring by hand. Despite these limitations, neurocognitive impacts of CS may be an unassessed mediator of HRQL results and long-term HRQL outcomes. In addition, a PerfO indicator of neurocognitive functioning would reduce confounds inherent in a PRO measure of cognition and could potentially yield valuable data to inform HRQL outcomes. Findings from this systematic literature review appear to lend support to a neurocognitive basis for neurocognitive and psychological functioning among CS patients, which may help explain the persistence of depression despite long-term remission.

### Measurement challenges in Cushing’s syndrome

A striking limitation across all 36 studies, including the four clinical trials and five longitudinal studies, is that no study captured a trajectory of HRQL change over three or more points of assessment. The clinical trials and longitudinal studies reported pre/post differences across varying durations of time, yet measuring HRQL across two time points can only reveal linear change. It is possible that CS patients experience linear or nonlinear HRQL trajectories over time, especially during the course of treatment.

An additional measurement limitation related to the limitations of the pre/post study design is that it does not provide for the statistical analysis of mediation in HRQL change over time [50]. Ideally, three or more assessment points over time would offer insight into the sequence of factors that influence changes in HRQL outcomes. Mediating factors, such as depression or BMI, may provide insight into direct and indirect mechanisms of change affecting HRQL trajectories for CS patients related to treatment outcomes.

The difficulties encountered in measuring PROs in rare diseases and the solutions developed to address them have been illustrated using CS as an example of rare disease. This specific example has provided us with elements found in other rare disease: the lack of a sufficient amount of studies, the use of generic PRO and other COA questionnaires, or the difficulty to have representative samples. Only recent similar initiative to address these issues can be found in the literature for other rare disease as well [51–53]. Validating a PRO instrument can be a difficult endeavour, as there are multiple psychometric dimensions to examine. For example, we have shown in this SLR that some studies provided clinical validity [27, 29], known-group validity [28, 34], and concurrent validity [31] for CushingQoL. The use of generic PRO and other COA questionnaires may be useful and easier when examining rare disease as they are readily available, however they may miss important specific data. A good solution to address this problem for CS was to use dedicated instruments, but some aspects of CS are not fully answered or validated, such as depression or cognitive symptoms. The representativeness and size of samples was also an issue raised in this SLR, and this is mostly due to the available population in rare diseases, which translates directly to a lower amount of studies. This can be a problem to really understand the outcomes in rare diseases as there are less evidence available. However, studies in CS showed clear significant link between change in physiological endpoints and PRO assessment, and this is partly due to the instruments developed either specifically for the study (event though not validated) [42], or for the rare disease (i.e., CushingQoL and Tuebingen CD-25).

This SLR highlighted HRQL measurement challenges, which were primarily related to limitations in study design and statistical analyses. Specifically, no study identified in this review involved three or more points of assessment, which limits the detection of trajectories of HRQL change over time and the analysis of mediating factors. For example, the postoperative glucocorticoid deprivation phase in the weeks or months after successful surgery worsens HRQoL measures due to extra pain, feeling of weakness, and other factors related to the sudden fall in cortisol exposure [54]. Additionally, evidence points to the possibility that HRQL subgroup differences may be present among CS patients (e.g., depression, neurocognitive functioning, BMI). This is probably because rare diseases’ population are usually heterogeneous (different symptoms, age, impact on quality of life, etc.). However, statistical analyses examined only mean group differences without examining the wide range of variance present in CS patient HRQL outcome data.

## Conclusion

CS is an iconic example of the difficulties encountered in measuring PROs in rare diseases. A solution for this specific case was developed in the form of dedicated PRO instruments, the CushingQOL and the Tuebingen-25. However, we have seen that some aspects of CS may not be fully answered or not yet validated (e.g., depressive and cognitive symptoms). As research progresses, new discoveries are made and neglected facets may be brought into light.

## Additional files

Additional file 1: Summary table type of PRO and study design per reference. A table summarizing the type of PRO and study design per reference. (DOCX 27 kb)

Additional file 2: Sub-domains of PRO assessed per PRO. A table summarizing the sub-domains of PRO assessed per PRO. (DOCX 18 kb)

## Abbreviations

BDI: Beck Depression Inventory
COA: Clinical Outcome Assessments
CS: Cushing’s Syndrome
EMA: European Medicines Agency
FDA: Food and Drug Administration
HRQL: Health-Related Quality of Life
MRI: Magnetic Resonance Imaging
PRO: Patient-Reported Outcome
QOL: Quality of Life
SLR: Systematic Literature Review
TMT: Trail Making Test
UFC: Urinary Free Cortisol
