# Supplementary material for: Illustration of patient-reported outcome challenges and solutions in rare diseases: a systematic review in Cushing’s syndrome
Source: Orphanet J Rare Dis. 2018 Dec 19;13:228. doi: 10.1186/s13023-018-0958-4 (PMC6299940; doi:10.1186/s13023-018-0958-4)
Supplement: Supplementary file 1 — Summary table type of PRO and study design per reference. A table summarizing the type of PRO and study design per reference. (DOCX 27 kb) [file 13023_2018_958_MOESM1_ESM.docx]

**Additional file 1:** Summary table type of PRO and study design per reference.

| Author year | CushingQoL | Tuebingen CD-25 | Other HRQOL measures | Study design |
| --- | --- | --- | --- | --- |
| Adelman 2013 | Yes | No | NR | Clinical trial |
| Andela 2015 | NR | NR | NR | Literature review |
| Badia 2013 | Yes | No | EQ-5D EQ-VAS | Retrospective study |
| Badia 2014 | Yes | Yes | NA | Literature review |
| Barahona 2010 | Yes | No | SF-36 | Retrospective study |
| Belaya 2015 | No | No | No | Retrospective study |
| Dorn 2000 | No | No | Wechsler Adult Intelligence Scale - Revised (WAIS-R) Profile of Mood States (POMS) SCL-90R | Prospective study |
| Fleseriu 2012 | No | No | BDI-II | Clinical trial |
| Huguet 2015 | Yes | Yes | BDI-II BDI SF-36 SCL-90-R HADS MFI-20 NHP SRT HADS-UK WHOQOL-BREF GHQ-8 FACT SAS-1 and 2 | Literature review |
| Iacobone 2012 | No | No | SF-36 | Prospective study |
| Iacobone 2012 (conference) | No | No | SF-36 | Prospective study |
| Katznelson 2012 | No | No | SF-36 | Clinical trial |
| Keil 2009 | No | No | The Child Health Questionnaire (CHQ) Wechsler Intelligence Scale for Children (WASI) CS symptom checklist | Prospective study |
| Keil 2013 | Yes | Yes | SF-36 | Literature review |
| Lindholm 2001 | No | No | SF-36 | Retrospective study |
| Llahana 2016 | No | No | NR | Literature review |
| Martinez-Momblan 2015 | Yes | No | Spanish Pain Questionnaire International Physical Activity Questionnaire (IPAQ) Oviedo Sleep Questionnaire (OSQ) Fagerstrom Test for Nicotine Dependence (FTND) Index of Erectile Function (IIEF 5) Female Sexual Functioning Inventory (FSFI)  and the Female Sexual Function Questionnaire brief profile (B- PFSF) Lifestyle Associated Questionnaire | Prospective study |
| Neychev 2015 | No | No | CS-specific Questionnaire | Retrospective study |
| Papakokkinou 2015 | No | No | MFS Comprehensive Psychopathological Rating Scale (CPRS-A) Neuropsychological Testing: TrailMaking Test A, B, C, D | Cross-sectional study |
| Papaoian 2014 | Yes | No | NR | Cross-sectional study |
| Papoian 2016 | Yes | No | No | Cross-sectional study |
| Pereira 2010 | No | No | *Starr, 1951 : Survey NR *Cohen, 1980: Interviews. Detailed clinical history and an examination of mental state *Sablowski et al., 1986: Freiburger Personality Inventory, Giessen test, State-Trait-Anxiety Inventory *Kelly et al., 1996: Present state examination, Hamilton rating scale Crown-Crisp experiential index, Eysenck personality inventory *Dorn et al., 1995: Interviews, atypical depression diagnostic scale, Hamilton rating scale, self-report instruments, medical records information *Dorn et al., 1997: Interviews, atypical depression diagnostic scale, Hamilton rating scale, self-report instruments, medical records information *Flitsch et al., 2000: Semi-structured interview, Freiburger Persönlichkeitsinventar, State-trait-anxietyinventory, Rosenzweig picture frustration test, Befindlichkeitsskala, Giessener Beschwerdebogen *Sonino, 2006: Tridimensional personality questionnaire, Symptom Rating Test | Literature review |
| Pikkarainen 1999 | No | No | *Visual Analogue Scale (VAS) *Working ability (no measured used) *Relapse in patients with pituitary disease (no measure used) | Retrospective study |
| Pivonello 2015 | ERCUSYN study: Yes | ERCUSYN study: No | NR | Literature review |
| Roset 2013 | Yes | No | SF-36 SF-6D | Cross-sectional study |
| Santos 2009 | Yes | No | EuroQoL-VAS (EuroQoL-5 Dimensions (5D) and its Visual Analogue Scale (VAS) | Cross sectional study |
| Santos 2014 | No | No | BDI-II STAI (State and Trait) | Cross-sectional study |
| Santos 2015 | Yes | Yes | SF-6D (derived from SF-36) SF-36 BDI Multidimensional body-self relations questionnaire Nottingham Health Profile HADS Multidimensionial Fatigue Index-20 RAND-36 | Literature review |
| Sippel 2008 | No | No | Questionnaire | Prospective study |
| Tiemensma 2011 | Yes | No | EQ-5D IPQ-R | Cross-sectional study |
| Tiemensma 2012 | Yes | No | EQ-5D SF-36 IPQ-R | Cross-sectional study |
| Tiemensma 2015 | Yes | No | NA | Cross-sectional study |
| Tiemensma 2016 | Yes | No | No | Cross-sectional study |
| Valassi 2011 | Yes | no | EQ-VAS | Prospective study |
| Valassi 2011 (conference) | Yes | No | EuroQoL | Prospective study |
| Wagenmakers 2012 | Yes | No | RAND-36 HADS Checklist Individual Strenght Questionnaire Cognitive Failures Questionnaire Appearance Self-Esteem Nottingham Health Profile | Cross-sectional study |
| Webb 2008 | Yes | No | Generic questionnaires | Cross-sectional study |
| Webb 2010 | Yes | No | No | Cross-sectional study |
| Webb 2012 | Yes | No | BDI | Clinical trial |

PRO: Patient-Reported Outcome.
