# Supplementary material for: Illustration of patient-reported outcome challenges and solutions in rare diseases: a systematic review in Cushing’s syndrome
Source: Orphanet J Rare Dis. 2018 Dec 19;13:228. doi: 10.1186/s13023-018-0958-4 (PMC6299940; doi:10.1186/s13023-018-0958-4)
Supplement: Supplementary file 2 — Sub-domains of PRO assessed per PRO. A table summarizing the sub-domains of PRO assessed per PRO. (DOCX 18 kb) [file 13023_2018_958_MOESM2_ESM.docx]

Additional file 2: Sub-domains of PRO assessed per PRO.

| PRO Name | PRO factors |
| --- | --- |
| CushingQOL | Areas assessed: trouble sleeping,  wound healing/bruising, irritability/moodswings/anger,  self-confidence,  physical changes, ability to participate in activities,  interactions with friends and family,  memory issues,  future health concerns |
| Tuebingen CD-25 | Areas assessed:  depression,  sexual Activity,  environment,  eating Behaviour, bodily Restrictions,  cognition |
| Short-Form 36 Health Survey ver2 (SF-36) | Not provided |
| SF-6D | 6 domains: physical functioning,  role limitation,  social functioning,  pain, mental health,  vitality.  Each domain has 4–6 levels of severity and an SF-6D health state is defined when a respondent selects one level of severity in each domain. |
| EuroQol 5D (EQ-5D) | Dimensions covered: mobility, self-care,  usual activities,  pain and discomfort,  anxiety depression |
| CS-specific Questionnaire | Cushing’s symptoms were stratified into three main domains (categories):  General Cushing’s Physical features (GCP) (4 items, ‘‘a’’ through ‘‘d’’);  Biochemical Abnormalities  Comorbidities (BAC) (4 items, ‘‘a’’ through ‘‘d’’);  Emotional-Behavioral features (EB) (4 items, ‘‘a’’ through ‘‘d’’). |
| EQ-VAS | Overall health status |
| Trail Making Test | Cognitive functioning |
| The Wechsler Adult Intelligence Scale–Revised (WAIS-R) | Cognitive functioning |
| Beck Depression Inventory (BDI)-II | Depressive symptoms |
| Profile of Mood States (POMS) | Depressive symptoms |
| Symptom Checklist 90–Revisited (SCL-90R) | Depressive symptoms were the focus of the study within this population |
| General health questionnaire | Psychiatric symptoms |
| Hospital Anxiety and Depression Scale | Anxiety and depression |
| Multidimensional Fatigue Inventory | Fatigue |
| Nottingham Health Profile | Sleep Mobility Energy Pain Emotional reactions Social isolation |
| Symptom Rating Test | Self-rating scale of psychological distress on 6 subscales:  anxiety,  depression, somatic symptoms,  anger-hostility,  cognitive symptoms  psychotic symptoms). |
| World Health Organization Quality of Life-BREF | Domains:  physical health,  psychological health,  social relationships,  environment. |
| Illness Perception Questionnaire - Revised | Identity Cause Timeline Consequences Cure-Control |
| Drawing Test | Not provided |
| Checklist Individual Strength Questionnaire | It consists of four dimensions: i) the subjective experience of fatigue, ii) reduction in motivation, iii) reduction in activity and iv) reduction in concentration |
| Cognitive Failures Questionnaire | everyday mistakes and problems regarding perception memory motor function orientation |
| Appearance Self-Esteem | Satisfaction with one's apperance |
| Child health questionnaire-parent report (CHQ) | Domains: physical (PhS) health- physical functioning, role/social functioning related to physical health, body pain/discomfort, and global health perception  psychosocial (PsS) health-social limitations related to emotional-behavioural health, self-esteem, mental health, general behaviour, emotional impact (parent), and time impact (parent).  Other-limitations in family activities and family cohesion |
| The Wechsler Intelligence Scale for Children | Cognitive functioning |
| CS Symptom Checklist (no official name was provided) | Signs and symptoms associated with CS |
| MFS (Mental Fatigue) | Mental fatigue |
| Comprehensive Psychopathological Rating  Scale (CPRS-A)35:3529:3429:3329:3229:31 | Anxiety and depression |
| Neuropsychological Testing: TrailMaking Test A, B, C, D | Speed of processing, sequence alternation, cognitive flexibility, visual search, motor performance, and executive function |
| Visual Analogue Scale (VAS) | Symptoms and quality of life |
